# Calcium-containing phosphopeptides pave the secretory pathway for efficient protein traffic and secretion in fungi

**DOI:** 10.1186/s12934-014-0117-0

**Published:** 2014-09-10

**Authors:** Juan F Martín

**Affiliations:** Área de Microbiología, Facultad de Ciencias Biológicas y Ambientales, Universidad de León, 24071 León, Spain

**Keywords:** Protein secretion, Casein phosphopeptides, Calcium binding, Calcium homeostasis, Secretory vesicles, Protein sorting, Protein targeting, Fusogenic activity, Casein kinase, Filamentous fungi

## Abstract

Casein phosphopeptides (CPPs) containing chelated calcium drastically increase the secretion of extracellular homologous and heterologous proteins in filamentous fungi. Casein phosphopeptides released by digestion of alpha − and beta-casein are rich in phosphoserine residues (SerP). They stimulate enzyme secretion in the gastrointestinal tract and enhance the immune response in mammals, and are used as food supplements. It is well known that casein phosphopeptides transport Ca^2+^ across the membranes and play an important role in Ca^2+^ homeostasis in the cells.

Addition of CPPs drastically increases the production of heterologous proteins in *Aspergillus* as host for industrial enzyme production. Recent proteomics studies showed that CPPs alter drastically the vesicle-mediated secretory pathway in filamentous fungi, apparently because they change the calcium concentration in organelles that act as calcium reservoirs. In the organelles calcium homeostasis a major role is played by the *pmr1* gene, that encodes a Ca^2+^/Mn^2+^ transport ATPase, localized in the Golgi complex; this transporter controls the balance between intra-Golgi and cytoplasmic Ca^2+^ concentrations. A Golgi-located casein kinase (CkiA) governs the ER to Golgi directionality of the movement of secretory proteins by interacting with the COPII coat of secretory vesicles when they reach the Golgi. Mutants defective in the casein-2 kinase CkiA show abnormal targeting of some secretory proteins, including cytoplasmic membrane amino acid transporters that in *ckiA* mutants are miss-targeted to vacuolar membranes.

Interestingly, addition of CPPs increases a glyceraldehyde-3-phpshate dehydrogenase protein that is known to associate with microtubules and act as a vesicle/membrane fusogenic agent. In summary, CPPs alter the protein secretory pathway in fungi adapting it to a deregulated protein traffic through the organelles and vesicles what results in a drastic increase in secretion of heterologous and also of some homologous proteins.

## Introduction

### What are casein phosphopeptides? Role in calcium transport in the intestinal tract

Casein phosphopeptides (CPPs) are bioactive peptides derived from casein which are rich in phosphoserine. Bioactive peptides were first discovered in milk, yogurt and casein during early studies on infant nutrition [1-3]. CPPs are present in milk from all studied mammalians but usually we refer to those obtained from bovine casein since they are the best known [4] and because they are available commercially [5]. In recent years they have been extensively studied [6,7].

The CPPs have gastrointestinal activities and enhance the immune response in rats and in humans. They are used as diet supplements for specific nutritional or medical uses in Japan and some western countries. Due to their high phosphoserine content CPPs form complexes with Ca^2+^ through ionic interactions and they provide increased calcium bioavailability by maintaining Ca^2+^-phosphopeptides in a soluble form and by favouring Ca^2+^ transport through the intestinal walls [8-10]. The degree of Ca^2+^ binding is directly related to the number of phosphoserine residues in each CPP.

The phosphoserine residues are partially hidden inside the globular casein alfa and beta molecules but they became exposed when they are released by tryptic digestion of casein [11]. There are several CPPs derived from casein but the best known are the so-called “β-casein 4P(1–25)” that corresponds to the N-terminal 25 amino acids of β-casein, “αS1 and αS2 casein 4-P (1–21)” and “αS2 casein 4-P (46–70)” (all of them with four phosphoserine residues) and “αS2-P (59–79)” containing five phosphoserine residues [10] . Studies of chemically synthesized peptides showed that the presence of the tripeptides serP-Leu-serP and/or serP-serP-serP stimulates proliferation of lymphocytes [12].

### Commercial phosphopeptides preparation and phosphopeptide enrichment and purification

Different degrees of purification of specific phosphopeptides are required for use as protein secretion enhancers in microbial fermentations or for the health and food-grade additives. Only for human medical applications, as inmunostimulants, may pure phosphopeptides be required.

Commercial preparations of CPPs in Japan and Europe are obtained by careful controlled hydrolysis of bovine casein with purified trypsin [13] or with successive digestions with pepsin and trypsin [11].

The available commercial preparations contain a mixture of phosphoserine-rich peptides including αS2 (1–21) (or 1–32, depending on the manufacturer) [10] and β − casein (1–25) and other poorly phosphorylated and unphosphorylated peptides. To increase the content of phosphoserine-rich peptides, CPPs preparations are enriched by several techniques [14].

### Phosphopeptides enrichment

As indicated above, for medical uses or for research purposes phosphorylated peptides need to be enriched and purified. Many proteins in all living beings are reversibly phosphorylated and the phosphorylation/dephosphorylation degree is an important regulatory parameter for these proteins [15,16]. In eukaryotic cells substrate proteins are mainly phosphorylated at the hydroxyl group of some serine, threonine and tyrosine residues by protein kinases and dephosphorylated by protein phosphatases. The interest in the identification of the phosphorylation site(s) of the proteins has led to the development of different procedures for enrichment of phosphorylated proteins and peptides (the so-called phosphoproteome). Most of these procedures rely on inmobilized metal affinity chromatography (IMAC) [17-19] or filtration through strong cation exchange resins and strong anion exchangers [20].

A simple enrichment procedure is to precipitate the phosphopeptides with calcium [14]. More recently an efficient phosphopeptide enrichment method has been reported that combines an step of precipitation with calcium phosphate followed by IMAC using either Fe(III)- or TiO2-inmobilized columns [21,22].

These techniques allow enrichment of phosphopeptides that may be later resolved by HPLC and characterized by high resolution LC/MS [23,24], coupled with dephosphorylation treatments with phosphatases if required to locate the position of the phosphorylated amino acids [23]. A mass (m/z) difference of 80 in the positive ion mode between the untreated and dephosphorylated peptide indicates the presence of a phosphate group in a CPP molecule.

### Fungi as host for secretion of homologous and heterologous proteins

Many proteins, particularly enzymes, need to be produced at large scale for the food and chemical industries and also for specific medical applications [25-27]. Several filamentous fungi, particularly members of the genus *Aspergillus* [28,29] and to a lower extent some species of *Trichoderma* and *Penicillium* (e.g. *Penicillium roqueforti*) are known to produce very large amounts of homologous proteins (up to 20 g/liter). This is the case of glucoamylase and α-amylase in *Aspergillus niger* [30]. Other related *Aspergillus* species include *Aspergillus awamori* [31,32] and *Aspergillus oryzae* [33]. For some applications in the food industry *Penicillium roqueforti, Penicillium nalgiovense or Penicillium chrysogenum* [34] may be also useful. All these fungi have been utilized extensively in food processiong and are considered to be GRAS (generally recognized as safe) organisms.

In addition to their high protein secretion capability filamentous fungi have other advantages as hosts for large scale production of enzymes, namely: i) fungi grow rapidly in a variety of culture media, ii) they produce correctly folded proteins, and iii) in fungi post-translational modifications of enzymes are similar to those that occur in animal cells. Also similar are the problems related to protein traffic through the vesicle-mediated system.

However, when animal or plant proteins (e.g. thaumatin) [21] or heterologous microbial proteins are expressed in the above mentioned fungal host strains, yields of the heterologous proteins are much lower than those of homologous proteins (amylases, proteases or lipases) in the same strains and are usually in the range of 10 to a few hundred mg of protein per liter of culture. The reason for this low yield is frequently the limitation of the mechanisms of protein folding and posttranslational protein modifications that are required for the protein to be transported by the organelle/vesicle-medianted transport in the secretory pathway. These limitations are bypassed (at least partially) by the action of the CPPs (see below).

The limitations observed in expression of heterologous genes have been widely addressed by several research groups including the use of strong constitutive and inducible promoters [27,30] and the construction of synthetic genes with optimal codon usage for *Aspergillus* [35-37] and is not further discussed.

### CPPs produce a drastic increase in the secretion of chymosin and other proteins in *Aspergillus*

During studies on the production of the milk-clotting enzyme chymosin in *Aspergillus*, using a synthetic *chy* gene (encoding the bovine chymosin) with optimized codon usage [35], it was found that the production of chymosin was highly dependent on the addition of casein to a defined medium even though the medium contained asparragine as nitrogen source and glucose and glycerol as carbon sources. Later, the stimulation by casein was shown to be due to the phosphopeptides present in the casein that are released by the *Aspergillus* proteases [38]. Several plant derived proteins (peptones) do not exert the stimulatory effect on chymosin production, although a clear stimulation was exerted by other animal protein, the bovine seroalbumin. The bovine casein and seroalbumin appear to be richer in phosphoserine peptides than plant-derived peptones.

The stimulatory effect of casein on chymosin production is not exerted by casein hydrolysates (casamino acids). This result might be explained by the different mechanisms of internalization of the phosphopeptides and the free amino acids (see below). Dephophorylated casein does not exert the stimulatory effect indicating that the drastic increase in chymosin production is due to the phosphorylated casein peptides. Interestingly pure phosphoserine does not produce any effect on protein secretion, in agreement with the observations of Otani et al. [12] on immunoglobulin production, suggesting that the stimulatory effect is due to the phosphorylated casein peptides and not to the free phosphoserine. Indeed, these authors [12] concluded that the presence of two or three adjacent (or separated by a single amino acid) phosphoserines in a peptide is required for the biological activity of CPPs.

The stimulatory effect of CPPs is not exerted at the transcriptional level. Northern analysis of expression of the *chy* gene in constructions using two different fungal promoters showed no changes in transcription of the *chy* gene following adition of CPPs. However, the increased secretion of this heterologous protein was confirmed by Western analysis using anti-chymosin antibodies and proteomic studies [38]. All the evidence available suggests that the stimulatory effect of CPPs is due to an activation of the vesicle-mediated transport system of secretory proteins.

### A global view of the secretory pathway: from the endoplasmid reticulum to the extracellular space

**Figure 1:**
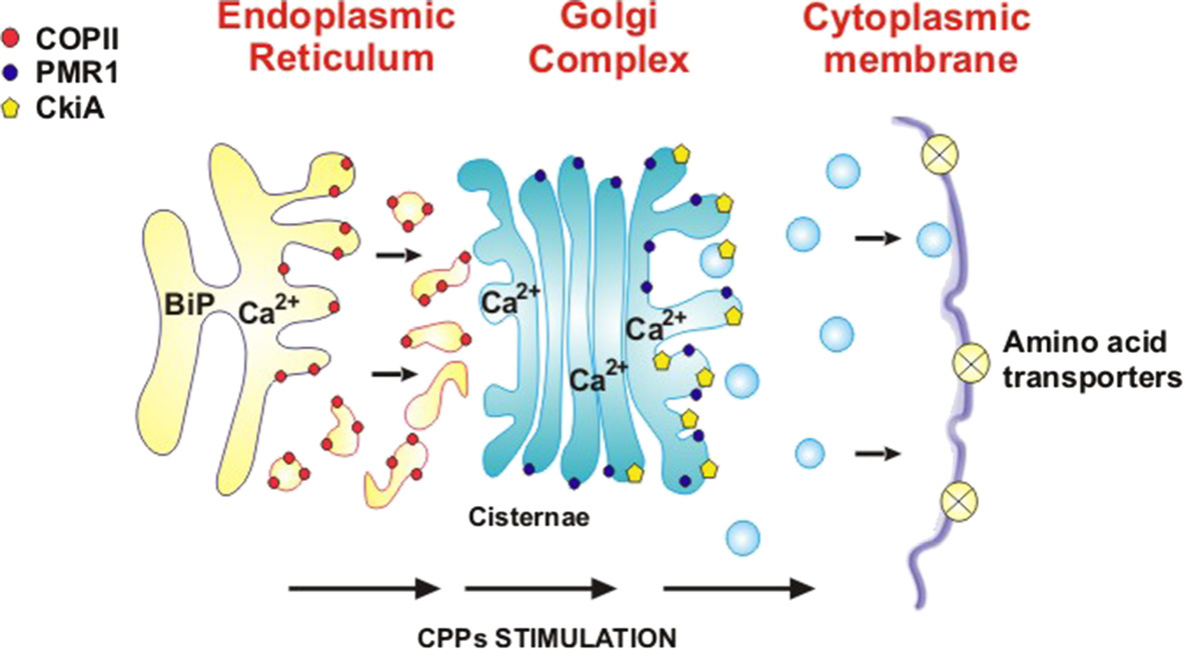
**Global view of the protein secretory pathway in filamentous fungi showing the location of the COPII complex in coated vesicles budding from the ER and merging to the Golgi complex where the CkiA (casein kinase) is involved in the vesicle coat removal and fusion of vesicles with the Golgi.** The PMR1 Ca^2+^/Mn^2+^ transporter ATPase is located in the Golgi membranes. Finally the amino acid permeases located in the cytoplasmic membrane are shown. The targeting of these amino acid permeases is governed by CkiA. The CPPs produce an overall stimulation of the forward traffic of secretory proteins by altering the Ca^2+^ concentration gradients in the organelles (solid arrow in the botton, see text for details).

The protein secretory route is a compartimentalized pathway [39] in which ribosomally synthesized proteins are finally targeted to the cell membrane and released to the extracellular space by an exocytosis mechanism, involving fusion of secretory vesicles to the cell membrane. The secretory proteins are transferred from one membrane enclosed organelle to vesicles and then to another organelle, and finally again through vesicles to the extracellular medium (Figure 1). The compartimentalization in different membrane-bound organelles or in vesicles serves to perform structural modifications of secretory proteins by modifiying enzymes residing in each compartment, thus avoiding competing modifying activities. Transfer of secretory proteins between organelles or between one organelle and the external cell space involves the packaging of appropriately folded, modified and oligomerized secretory proteins into distinct types of membrane-bound vesicles that ferry the proteins to different target sites.

The secretory protein pathway in yeast and filamentous fungi is similar in many respects to the protein secretion system in animal cells except that in filamentous fungi protein secretion is concentrated in the region near the tip of the hyphae. In the first step proteins containing a signal peptide at their N-terminal end [40,41] are recruited and transported into the endoplasmic reticulum (ER), concomitantly with cleavage of the signal peptide, by resident signal peptidases. In the ER the secretory proteins are adequately folded and modified by glycosylation and phosphorylation with the help of chaperones and foldases (such as BiP) and glycosylating and phosphorylating enzymes.

### The protein-Ca^2+^ gel: a role of BiP and Ca^2+^ on folding and packaging of secretory proteins

One of the ER-resident proteins, the chaperone BiP, has a Ca^2+^ binding activity and binds also to the nascent polypeptides being inserted into the ER lumen. Due to these activities BiP binds simoultaneously to the incompletely folded nascent proteins in the ER and to calcium, forming a gel, together with the other Ca^2+^-binding ER-resident proteins. The nascent proteins in the gel matrix are retained in the ER-lumen allowing them to be properly folded.

The Ca^2+^-protein gel binds to the phospholipids in the ER stabilizing the membrane and preventing undue vesicle budding from the ER. CPPs contain Ca^2+^ and bind to phospholipids [9], and they may act also in modulating the vesicles stability. When the nascent protein is properly folded and processed, the BiP protein is released from the protein-Ca^2+^ gel, and specific local areas of the ER membrane are desestabilized allowing vesicle budding (Figure 1). The properly folded nascent protein is packaged in vesicles and ferried to the Golgi complex or to alternative destinations in other membrane-bound systems in the cell, including the cytoplasmic membrane.

After leaving the ER the proteins are chanelled to the cisternae of the Golgi complex. The use of protein labeled with green or red fluorescent proteins (GFP or DsRed) combined with cnfocal fluorescence microscopy has allowed tracking of the proteins during transit from the ER to Golgi. Contrary to initial belief on the packaging in spherical vesicles, it has been evidenced that the secretory proteins are trapped in tubular structures that move directionally along actin microtubules following curvilinear paths. These pre-Golgi tubular cisternae finally are converted to the true Golgi complex. Some additional protein modifications occur in the Golgi complex, particularly extension of the carbohydrate chains or trimming down of the glycoproteins sugar chains.

### Secretory chekpoints

#### CPPs cause a checkpoint bypass

The secretory proteins in the ER are folded with the help of the calcium-binding protein BiP [42]. Each packaging step into different vesicles serves as a checkpoint of quality control of the secretory proteins. Only those proteins that are adequately folded, oligomerized and processed according to the specific requirements of each class of carrier vesicles are packaged. Other proteins will be retained for refolding or, alternatively, targeted to vacuoles for recycling or degradation.

The packaging of the secretory proteins when leaving the ER is considered to be a major checkpoint since not all ER proteins are allowed to proceed to the Golgi complex. Traffic of proteins in vesicles from the ER to the Golgi complex requires GTP and Ca^2+^ ions. Post-Golgi checkpoints are also likely to exist since mutants in late steps of the secretory pathway are known [43] that alter the secretion process.

The level of Ca^2+^ in the cytoplasm varies depending on several nutritional parameters and appears to play an important role in driving the movement of secretory vesicles through the hyphae [44,45]. In yeast and mammalian cells the ER acts as a Ca^2+^ reservoir. There are four Ca^2+^ -binding proteins (one of them BiP) that maintain a high Ca^2+^ concentration (up to three orders of magnitude) inside the ER. This concentration of Ca^2+^ in the ER is created by a membrane ATPase that stablishes an ion gradient across the membrane using ATP. The stored Ca^2+^ can be released by the action of inositol-triphosphate [*ins*(1,4,5)-P3]. This compound is considered to be the controller of the organelle Ca^2+^ reservoir under normal physiological conditions [46].

Interestingly, the calcium concentration in the cytoplasm and the ER can also be modified by calcium ionophores independently of the role of *ins*(1,4,5)-P3. Calcium ionophores or mutations that impair the distribution of intracellular Ca^2+^ alter the retention of ER-resident proteins, such as GRP94, and allow indiscriminated secretion of ER resident and normal secretory proteins. One yeast mutant altered in the *PMR1* gene, which encodes a Ca^2+^ ion transporter, shows poor growth in Ca^2+^-limited medium and is sensitive to the Ca^2+^-chelating agent EGTA [47]. In this mutant ER-resident proteins are packaged together with normal secretory proteins resulting in abnormally increased protein secretion. Even incompletely glycosylated proteins that normally would be retained in the ER, are secreted in this mutant. This evidence led Sambrook [39] to propose that alteration of Ca^2+^ levels in the organelles versus the cytoplasm is the signal that triggers formation of secretory vesicles and their transport to the final destination. If the Ca^2+^ concentration change is too drastic, abnormal packaging of both secretory proteins and ER-resident proteins occurs. As mentioned above CPPs contain chelated Ca^2+^ and introduce this ion through the cell membrane [8] and they likely alter the intracellular Ca^2+^ concentration, producing an effect similar to that of PMR1 mutations in *Saccharomyces cerevisiae*.

The BiP protein (GRP78 in mammals) is an ER-resident calcium binding protein that responds to the intracellular Ca^2+^ ion concentration and also to the level of unfolded or malfolded protein (triggering the so-called unfolded protein response, UPR) [48]. Both Ca^2+^ and the unfolded protein levels regulate expression of the *bipA* gene through binding of the C1 transcriptional factor. This factor recognizes a GGAGG sequence adjacent to the CCAAT promoter region. Binding in vitro of the C1 factor to the GGAGG sequence decreases in the presence of high Ca^2+^ concentration and therefore, expression of the *bipA* gene is increased at lower intracellular Ca^2+^ concentration [49].

### The *PMR1* protein of fungi: a Ca^2+^/Mn^2+^ ion pump involved in the secretory pathway, located in the Golgi and Golgi-like structures

The early work of Rudolph et al. [47] established that the previously sequenced yeast gene named *PMR1*, that affected the secretion of extracellular proteins was a Ca^2+^ ion pump similar to those of mammalian cells. Deletion of the *PMR1* gene in *S. cerevisiae* resulted in mutants that are unable to perform the protein outer glycosylation that is usually made in the Golgi.

*S. cerevisae* does not secrete efficiently heterologous mammalian proteins such as urokinase and bovine chymosin, apparently because these proteins are not properly modified and folded. Interestingly, yeast *PMR*1 mutants secrete these heterologous proteins more efficiently and unspecifically [50]. Also the endogenous yeast invertase, a glycosylated enzyme is more efficiently secreted in the *PMR*1 mutants, although the secreted invertase lacks the branched mannose outer residues that are added during passage through the Golgi. These results suggest that alteration of the *PMR*1-encoded Ca^2+^ ion-pump results in incompletely glycosylated proteins and more efficient packaging and secretion of these incompletely glycosylated forms.

Antebi and Fink [50] using cell fractionation studies observed that the bulk of Pmr1 protein co-localizes with Golgi markers (Figure 1) but a fraction of it was proposed to be located in Golgi-like structures. Null *PMR*1 mutants showed growth deffects which were suppressed by millimolar concentrations of Ca^2+^. This led to propose that *PMR*1 encodes a Ca^2+^ ion pump. Later Dürr et al. [51] observed that the growth defects of *PMR*1 mutants were also reversed by addition of Mn^2+^, suggesting that this gene encodes a dual Ca^2+^/Mn^2+^ pump that accumulates these ions in the Golgi and Golgi-like complexes. Okorokov and Lehle [52] confirmed that the *PMR*1 protein localizes in the Golgi but observed that null *PMR*1 mutants still contain 50% of a *PMR*1-like activity. They concluded that there is a second Ca^2+^ ion transporter located in the ER and observed considerable redistribution of membrane located enzyme activities in the *PMR*1 mutants [53]. Mutations of the *PMR*1 gene and functional analysis of the mutant proteins suggested the involvement of some amino acids side chains of one transmembrane spanning domain (TMS-6) in binding Ca^2+^ ions [54,55].

The PMR1 protein is a member of the SPCA (secreting pathway calcium ATPases family) that delivers Ca^2+^ ions to the intracellular organelles, particularly to the Golgi compartment [47,50,51,53,54,56].

Based on the yeast evidence recent studies have focused on the role of the fungal *pmr1* gene in Ca^2+^ homeostasis. This ion has an important role on the hyphae filamentous type of growth [57-60]. Recently Bowman et al. [61] have characterized two different mutants of the model fungus *Neurospora crassa*, one of them (Δ*pmr1*) completely deleted in the *pmr1* gene and the other containing specific nucleotide changes in this gene. Both mutants showed poor growth with highly branched hyphae and required Ca^2+^ and Mn^2+^ for optimal growth. The Δ*pmr1* mutant showed a drastic reduction (of 80%) in the accumulation of intracellular Ca^2+^ as compared to the wild type parental strain. The morphological defects could be partially reversed by Mn^2+^, although this ion did not affect the reduction of Ca2+ in the mutant, suggesting that the morphological effects caused by both Ca^2+^ and Mn^2+^ [62] are different from the specific effects on protein secretion caused by changes in calcium homeostasis.

The *N crassa* Pmr1 Ca^2+^ ATPases belong to the PMCA subfamily (plasma membrane Ca^2+^ ATPases) of secretory ATPases. In the genomes of different filamentous fungi; there are from two to six Ca^2+^ ATPases that are located in the ER, the Golgi, the vacuolar membrane or other membrane systems [61-63].

Studies on the *pmr1* gene of *Aspergillus fumigatus* [64] revealed similar effects of the *pmr1* mutation, resulting in slow growth and high branching of hyphae; these defects were suppressed by supplementing the medium with a high Ca^2+^ concentration. In contrast, the *pmr1* mutant of *Aspergillus niger* has no significant effect on morphological differentiation of this fungus, although growth of the mutant was stimulated by adding calcium [65,66]. Further research is needed to clarify possible differences in the control of calcium homeostasis between different filamentous fungi [66,67].

Tha addition of CPPs to *A. awamori* cultures mimics the secretion-enhancing effect of *pmr1* mutations, suggesting that CPPs act by altering the Ca^2+^ homeostasis in the cells.

### COPII, a complex of proteins forming the vesicle coat is involved in ER to Golgi protein traffic

During the decades from 1990 to 2010 a series of important advances in our understanding of vesicle-mediated protein traffic were made. They contributed to the understanding of the enhanced secretion of extracellular proteins [68]. Using in vivo studies with yeast mutants and in vitro reconstitution experiments with cell fractions, several research groups contributed significant advances [69,70]. Based on genetic evidence and reconstitution experiments Seaman and Robinson [71] showed the involvement of a vesicle coat formed by Sec proteins that is required for vesicle buding from the ER. It was named COPII (coat complex II) to distinguish it from the COPI complex involved in vesicle formation at the Golgi.

Mossessova and coworkers [72] proved that the nucleotide GTP is required for vesicle formation due to the involvement in COPII of the small GTP-binding protein Sar1p. The COPII complex is assembled at the ER when the activated form of the GTPase Sar1p is recruited by the Sec23p/sec24 proteins as Sar1p-GTP interacts with the sec23p and the GTPase-activating protein (GAP). Polymerization of the coat requires proteins Sec13p/sec31p (outer shell proteins) that are recruited by interaction with the sec23p/sec24p complex. The initial interaction of a coated vesicle with the target membrane is mediated by a class of proteins named “tethers” that work together with the Ras1 family GTPase. The tethering factor TRAPP1 is a protein that binds and activates the Ras1 GTPase Ypt1p [73,74].

### Interaction of the TRAPP1 factor with the sec23p of the membrane and a casein kinase determines the direction of vesicle traffic

The directionality of the traffic from ER to Golgi (forward traffic) or from Golgi to ER (backward or retrograde movement) has remained obscure for years until Lord et al. [75] established that the involvement of a casein kinase Hrr25p is essential for the directionality of the vesicle traffic. A decrease of the Hrr25 casein kinase activity in a yeast mutant suppreses the vesicle budding deffect in this mutant [76]. Interestingly this casein kinase, located exclusively in the Golgi, upon vesicle arrival to the Golgi, displaces the TRAPP1 factor from the coat Sec23p and phosphorylates the Sec23p/Sec24p proteins of the vesicle coat [75]. The unmasked COPII in the coated vesicle is then allowed to pair with the target (t-SNARE) docking protein (Sec22p) in the Golgi to discharge its cargo content. The sequential binding to COPII of Sar1p, then of TRAPP1 and finally the interaction with the casein kinase assures the forward directionality of the vesicle traffic and prevents the retrograde movement and the fusion of the vesicle back to the ER, since the casein kinase is located in the Golgi. Indeed, fluorescent protein targeting analysis of the Hrr25p casein kinase revealed that most of it (95%) co-localizes with pre-Golgi (punctuated particles) and late Golgi protein markers.

The Hrr25 kinase belongs to a family of casein kinases (Cki), that phosphorylates casein and other proteins at serine/threonine residues and its activity has implications on membrane protein traffic [77,78]. The casein kinase Hrr25p has an orthologue CK1p in the Golgi of mammals that is also involved in the vesicle traffic from ER to Golgi [77].

Apostolaki et al. [79] reported that three different strains of *Aspergillus nidulans* that were initially isolated as defective in amino acid transport and resistance to amino acid analogues were indeed mutants defective in the Hrr25p homologue casein kinase (named CkiA in *A. nidulans*). These mutations in the CkiA gene correlated with a rerouting of two membrane amino acid transport proteins (AgtA for glutamate and PrnB for proline). In the CkiA mutant these transporter proteins are targeted to the vacuoles instead of the cytoplasmic membrane and the absence of these transporters in the cell membrane explains the inability of the mutant strains to transport and utilize these amino acids or their toxic analogues.

The amino acid transporter proteins that are re-directed to the vacuole belong to a well characterized family of amino acid transporters all of which share a topology containing 12 transmembrane spanning domains (TMS). The highly conserved structure of those transporter proteins suggests that this topology may have a role in the passage of these transporters through the ER and Golgi and in their final insertion in the cell membrane [79].

The Cki homologue Hrr25p in *S. cerevisiae* is known to phosphorylate the sec23p component of COPII [75]. It seems unlikely that the *Aspergillus* casein kinase activity modifies the mechanism of the COPII-mediated ER to Golgi directionality of secretory protein traffic that seems to be conserved in different eukaryotic cells. Rather, the phosphorylation leads to redirecting the destination of the membrane targeted proteins [79]. Similarly, in the light of present evidence the casein phosphopeptides are likely to be protein traffic signals that mimic the role of peptide substrates of the casein kinase in protein traffic (see proteomic studies below).

### Physiological and proteomic studies on the effect of CPPs

In filamentous fungi growth takes place by hyphal tip extension. The hyphal tip is packed with vesicles and some membrane enclosed organelles, and it is known that cell wall biosynthetic enzymes and other nutrient hydrolytic enzymes are secreted at the tip or in the region near the tip [80]. In *N. crassa* a Ca^2+^ gradient is known to occur around those organelles [44,45].

Early physiological studies related to the nutritional role of CPPs revealed that CPPs play an important role on the regulation of gastric enzyme secretion in young mammals [8,12,13,81-84]. These effects were associated with changes in Ca^2+^ transport in the gastrointestinal tract cells [9]. CPPs also trigger the secretion of lynfokines in epithelial cell cultures [7].

Recently proteomics studies using cultures of *A. awamori* showed that supplementation with CPPs resulted in a drastic increase in the secretion of cell-wall synthesizing enzymes and several other extracellular enzymes which are known to be secreted through the vesicle-mediated secretory pathway [38]. The secreted cell-wall synthesizing enzymes include two different glucanosyltransferases and a GPI-linked cell wall polymer-organizing protein. The extracellular enzymes include α-amylase, glucoamylase, β-galactosidase, several proteases of *Aspergillus* and the engineered calf chymosin cloned into the fungal host. The secreted chymosin is increased 6.5-fold in CPP supplemented cultures with respect to the unsupplemented ones. The increased secretion of proteins is accompanied by a sharp decrease in the intracellular concentration of the precursor proteins of the secretory enzymes. Even ER-resident proteins such as BiP and other related chaperones of the heat-shock family (Hsp70 family) are depleted in CPPs supplemented cultures. Also an Hsp90 co-chaperone and a cyclophilin-like peptidyl-prolyl cis-trans isomerase that contributes to protein folding, are partially or totally depleted in the cells following CPPs addition. The same is true for the lectin chaperone calnexin. The underrepresentation of the chaperones in the intrancellular proteome of CPP-supplemented cells is probably due to the switch-off of the unfolded protein response (that in unsupplemented control cultures triggers the synthesis of BiP and other chaperones, [85]) since incompletely folded proteins are no longer retained in the ER.

### A key role for the protein glyceraldehyde-3-phosphate dehydrogenase induced by CPPs

One of the overrepresented proteins in the proteome of CPPs-supplemented cells is a glyceraldehyde-3-phosphate dehydrogenase (GPDH). This protein, in addition to its well known role in glycolysis, has vesicle fusogenic activity, microtubule bundling and a microtubule phosphotransferase/kinase activity that appears to be related to Ca^2+−^gradient during vesicle-mediated transport of secretory proteins [86-89]. This protein bundles microtubule in brain cells [87].

This GPDH has kinase/phosphotransferase activity. It is known that it autophosphorylates and also phosphorylates proteins in microsomes of skeletal nuucle cells [88]. GPDH has a Ca^2+^-dependent vesicle fusogenic activity in human neutrophils [89] and is involved in vesicle transport of secretory proteins during early transport of secretory proteins from ER into the Golgi complex [90,91].

Since *A. awamori* cells supplemented with CPPs show a high level of GPDH (38), the stimulation of vesicle transport and fusogenic activity of GPDH is probably one of the major effects of CPPs that that explains the enhanced protein secretion.

### From Golgi to the cell membrane and the extracellular medium

Filamentous fungi grow by apical extension at a relatively high speed (up to 0.5 μm per min) [92]. This implies that cell-wall precursors, cell wall synthesizing enzymes and other secretory proteins have to move relatively long distances in the hyphae [93] since it is well known that secretion of those enzymes concentrates at the apical and subapical regions [94-97]. Movement of the cargo-loaded vesicles proceeds along microtubule tracks [92] until it reaches the end of the microtubule where they merge with the cell membrane releasing their protein cargo.

### Endocytosis and possible internalization of CPPs

The microtubule-guided secretion is coupled to endocytosis, a compensatory mechanism by which external peptides and proteins are engulfed by membrane fragments which are excised from the cell membrane. Following internalization, the endosomes and their protein/peptide content play an important role in vesicle-membrane traffic in eukaryotic cells. Although endocytosis in fungi may occur in different locations in the hyphae all evidences suggest that it takes place largely in a subapical region, forming a characteristic endosome collar near the hyphae tip [98].

The mechanism(s) of CPPs internalization is still unknown; these compounds are highly charged. If casein phosphopeptides are taken up by endocytosis, their drastic effect on vesicle-mediated protein secretion can be easily explained.

During endocytosis the initial early endosomes move from the cell membrane to the nuclear periphery and this “centripetal” movement is in equilibrium with the microtubule-mediated “centrifugal” vesicle movement that is consistent with protein secretion. Whereas the centripetal movement is dynein-dependent the centrifugal motility along microtubules is regulated by kinesin.

It is likely that internalized CPPs play a role in the regulation of kinesin and/or dynein, thereby driving the vesicles towards the cell membrane [94,99,100]. This model is consistent with the phosphorylating role of GPDH and with recent findings on membrane organelle distribution [101] in fungal cells [102,103].

#### Future perspectives

It is clear from the available data that secretion of heterologous proteins in *S. cerevisiae* is inefficient [104]. Addition of casein phosphopeptides has a dramatic effect on secretion of homologous, and particularly of heterologous proteins, in filamentous fungi. This evidence is reinforced by recent studies on proteomics of unsupplemented and CPPs-supplemented fungal cells. Supplementation leads to a drastic decrease of the intracellular content of secretory proteins resulting in a large increase of the extracellular forms of these proteins.

Casein phosphopeptides contain chelated Ca^2+^ ions and interact with membrane phospholipids. Although their internalization mechanism is unknown it may involve endocytosis [105]. One of the proteins overrepresented in CPPs-supplemented cells is glyceraldehyde-3-phosphate dehydrogenase that has microtubule binding and vesicle-fusogenic activity. The enhancement of these activities as a response to CPPs addition explains the increased secretory vesicle traffic. CPPs seem to alter the distribution of intracellular membrane organelles favouring the forward (anterograde) movement of cargo loaded vesicles from ER to Golgi and then from Golgi to the cell membrane.

The vesicle movement guided on microtubules tracks is a likely target of regulation by Ca^2+^ and CPPs but more research is needed to clarify the molecular mechanisms of this regulation. Also a comprehensive understanding of the modification of Ca^2+^ levels in the organelles is needed.

Independent of the molecular mechanisms, inexpensive CPPs may be easily used to increase the production of homologous and heterologous proteins in filamentous fungi in cell factory processes. Optimization of the fermentation parameters in each case will help to improve the titers of the secreted proteins.

## Abbreviations

CPPs: Casein phosphopeptides
SerP: Phosphoserine
GPDH: Glyceraldehyde-3-phosphate dehydrogenase
ER: endoplasmic reticulum
BiP: Binding protein (to unfolded nascent polypeptides)
